# User Perceptions of Visual Clot in a High-Fidelity Simulation Study: Mixed Qualitative-Quantitative Study

**DOI:** 10.2196/47991

**Published:** 2024-01-11

**Authors:** Greta Gasciauskaite, Clara Castellucci, Amos Malorgio, Alexandra D Budowski, Giovanna Schweiger, Michaela Kolbe, Daniel Fries, Bastian Grande, Christoph B Nöthiger, Donat R Spahn, Tadzio R Roche, David W Tscholl, Samira Akbas

**Affiliations:** 1 Institute of Anesthesiology University Hospital Zurich Zurich Switzerland; 2 Simulation Centre University Hospital Zurich Zurich Switzerland

**Keywords:** Visual Clot, blood coagulation, blood coagulation test, hemostasis, rotational thromboelastometry, situation awareness, user-centered design, visualization, user, perception, interpretation, thromboelastography, viscoelastic hemostatic, technology, coagulation, quantitative information

## Abstract

**Background:**

Viscoelastic hemostatic assays, such as rotational thromboelastometry (ROTEM) or thromboelastography, enable prompt diagnosis and accelerate targeted treatment. However, the complex interpretation of the results remains challenging. Visual Clot—a situation awareness-based visualization technology—was developed to assist clinicians in interpreting viscoelastic tests.

**Objective:**

Following a previous high-fidelity simulation study, we analyzed users’ perceptions of the technology, to identify its strengths and limitations from clinicians’ perspectives.

**Methods:**

This is a mixed qualitative-quantitative study consisting of interviews and a survey. After solving coagulation scenarios using Visual Clot in high-fidelity simulations, we interviewed anesthesia personnel about the perceived advantages and disadvantages of the new tool. We used a template approach to identify dominant themes in interview responses. From these themes, we defined 5 statements, which were then rated on Likert scales in a questionnaire.

**Results:**

We interviewed 77 participants and 23 completed the survey. We identified 9 frequently mentioned topics by analyzing the interview responses. The most common themes were “positive design features,” “intuitive and easy to learn,” and “lack of a quantitative component.” In the survey, 21 respondents agreed that Visual Clot is easy to learn and 16 respondents stated that a combination of Visual Clot and ROTEM would help them manage complex hemostatic situations.

**Conclusions:**

A group of anesthesia care providers found Visual Clot well-designed, intuitive, and easy to learn. Participants highlighted its usefulness in emergencies, especially for clinicians inexperienced in coagulation management. However, the lack of quantitative information is an area for improvement.

## Introduction

Rapid hemostatic assessment is essential to targeted coagulation management in acute bleeding [1]. Increasingly used viscoelastic hemostatic assays, such as rotational thromboelastometry (ROTEM) or thromboelastography, enable faster insights into coagulation dysfunction than conventional laboratory tests. Standard coagulation assays are not optimal for managing acute hemorrhages that require rapid therapeutic action, as it often takes more than an hour to obtain the results [2,3]. European and North American transfusion recommendations underline the advantages of viscoelastic hemostatic assays for managing trauma and severe perioperative bleeding, including the reduced need for transfusions, fewer perioperative complications, shorter hospitalization, and lower overall treatment costs [1,4,5]. Its usefulness has been demonstrated in many operative areas such as obstetrics [6,7], pediatric surgery [8], transplantation [9], cardiac surgery [10,11], neurosurgery [12], and burn surgery [13,14]. Viscoelastic hemostatic tests are also paramount in the diagnostic and treatment adjustment of hematological disorders, such as inherited afibrinogenemia, hemophilia, or multiple myeloma [15-18]. However, despite these technologies’ widespread use and considerable advantages, their results’ interpretation remains challenging and requires well-trained clinical personnel [19-21]. Visual Clot—a situation awareness-based visualization technology—was developed to support health care professionals in interpreting viscoelastic test results by reducing the complexity of their presentation. Based on raw ROTEM data, the results are displayed in real time as a 3D animated model of a blood clot to represent various elements of hemostasis, including platelets, plasmatic factors, and fibrin. It can also effectively illustrate the influence of heparin and hyperfibrinolysis [22] (Multimedia Appendix 1). In a high-fidelity simulation study, anesthesiologists using Visual Clot were 2.2 times more likely to articulate the correct therapeutic approach. In addition, these anesthesiologists had a lower median time to administer the first appropriate targeted coagulation product. Overall, physicians presented with the results of viscoelastic testing using Visual Clot were approximately 56% more likely to provide accurate therapeutic interventions. In the same study, physicians were 3.5 times more likely to feel confident in their decisions when working with Visual Clot compared to traditional ROTEM results [23]. In the first computer-based study analyzing user perceptions of Visual Clot, participants described the technology as well-designed, easy to learn, and intuitive [24]. The guiding principles of the Visual Clot technology that result in enhanced situation awareness include Endsley’s user-centered design principles [25], Wittgenstein's philosophy as articulated in Tractatus Logico-Philosophicus [26], and insights from the National Aeronautics and Space Administration (NASA) publication “On Organization of Information: Approach and Early Work” by Degani et al [27]. Endsley’s principles emphasize the use of direct visual representations of data to enhance situational awareness, a central principle in Visual Clot's data visualization. Wittgenstein's theory emphasizes the importance of logical representations that meaningfully correspond to the reality they are intended to represent. Visual Clot follows this principle by visually representing elements such as fibrin, platelets, plasmatic factors, hyperfibrinolysis, and bleeding. Following NASA's approach, Visual Clot strives to achieve the highest level of “order and wholeness” by consolidating all essential data into a single display. The primary goal of Visual Clot technology is to provide the care provider with situational information quickly and with minimal cognitive load. In this study, we aimed to capture and analyze perceptions of anesthesia personnel working with Visual Clot in a high-fidelity simulation to identify the strengths and recognize the potential for future improvements.

## Methods

### Ethical Considerations

The Cantonal Ethics Committee of the Canton of Zurich reviewed the study protocol and issued a declaration of no objection (Business Management System for Ethics Committees Number Req-2021-01112). Furthermore, each participant gave informed consent to use his or her data for research purposes. Participation was voluntary and without financial compensation.

### Study Design

We conducted a researcher-initiated single-center mixed qualitative-quantitative study at the University Hospital Zurich, Institute of Anesthesiology, Switzerland. Study participants were anesthesia personnel, including staff anesthesiologists, residents, and nurses. After participating in a high-fidelity simulation study of perioperative bleeding scenarios, where they worked with Visual Clot and ROTEM, we interviewed participants on their perceptions of Visual Clot technology.

As a second step, the same participants received an email invitation to participate in a survey a few weeks later. They rated statements we generated from identified and frequently mentioned themes in interview responses on a Likert scale.

### Previous High-Fidelity Simulation Study

In the high-fidelity simulation study [23], anesthesia teams, composed of a staff anesthesiologist, a resident, and an anesthesia nurse, participated in high-fidelity perioperative bleeding scenarios using either Visual Clot or ROTEM. The primary outcome of the study was correct targeted coagulation therapy. Secondary outcomes were time to targeted coagulation therapy, confidence, and workload.

ROTEM is the standard of care for managing acute hemorrhage in the study center, so all participants were familiar with the technology before participating [20]. Some participants had taken part in previous Visual Clot studies and, therefore, were already familiar with the technology [22,24].

**Figure 1:**
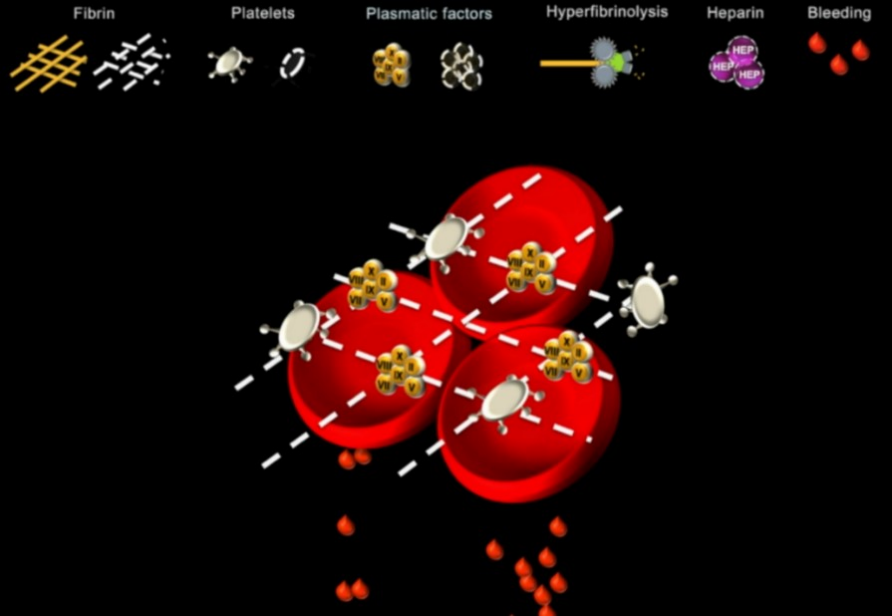
A Visual Clot result presentation showing a fibrin deficiency. The fibrin in the clot is shown as a dashed line, indicating its absence. The blood drops indicate the presence of a coagulation pathology.

Nevertheless, before the simulations began, we gave a 10-minute presentation that reviewed ROTEM and introduced Visual Clot. Multimedia Appendix 1 provides an instructional video of Visual Clot. Participants were invited to ask questions freely before starting work in the simulation environment. Each team solved 1 of 4 different perioperative bleeding scenarios, which were randomly allocated. We ended the scenarios when all necessary therapeutic measures were derived or, at the latest, after 15 minutes. Figure 1 illustrates an example of a Visual Clot printout used in the simulation study.

### Participant Interviews

After the simulations, we encouraged the participants to freely verbalize their thoughts in a distraction-free environment while the data collectors made field notes. The only suggestion to the participants before the interviews was to verbalize their positive and negative opinions of Visual Clot. The participants could define final adjustments in the collected answers at the end of the interviews.

### Survey

In the second step, we formulated 5 statements to summarize the insights gathered during the interviews. The statements were submitted for evaluation on a 5-point Likert scale graded from “strongly agree” to “strongly disagree.” An email invitation was sent to all interviewed participants.

### Outcomes and Statistical Analyses

#### Part I: Participant Interviews

Collected interview responses were translated from original German to English using a translation system DeepL (DeepL GmbH). Multimedia Appendix 2 provides the complete translated field notes.

**Figure 2:**
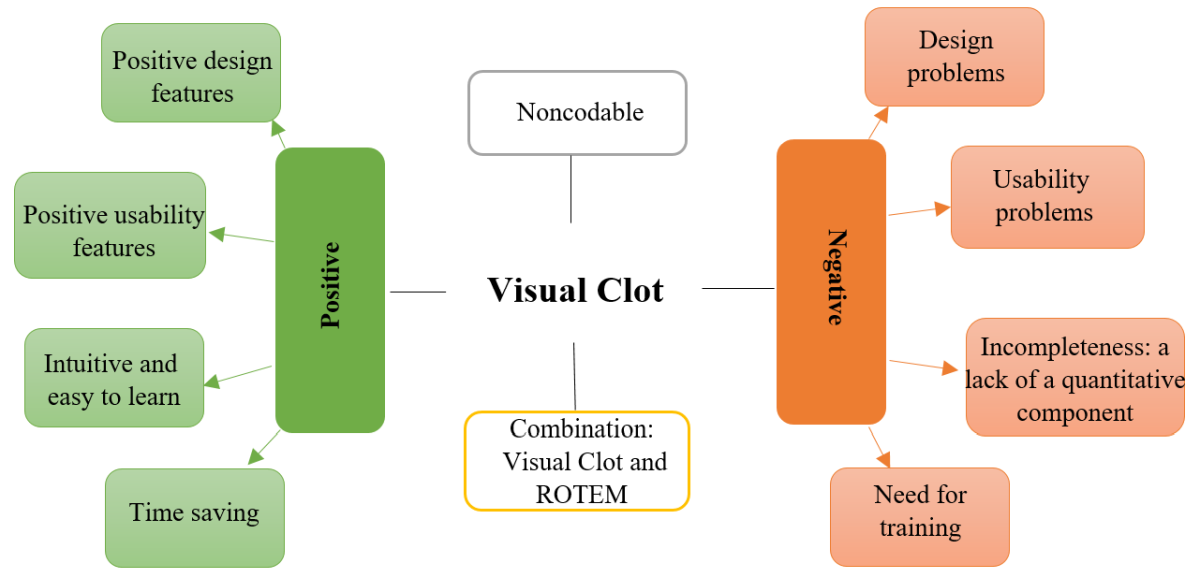
A coding tree representing the themes describing positive and negative user perceptions. ROTEM: rotational thromboelastometry.

The most commonly used terms in positive and negative responses were identified using the word count function. Word groups with the same root were united, excluding the frequently used filler words such as “to,” “and,” or “the” (Table 1). Using a template approach [28] we identified the major themes that dominated participants’ answers. As a result, we generated a coding tree (Figure 2). According to the coding template, we assigned statements to the themes. A total of 3 of the study authors, all anesthesiology residents GG, GS, and SA, rated the interview statements separately from each other using the coding tree (Figure 2). If the 3 investigators disagreed after multiple data coding, the final decision was taken in a joint discussion. Interrater reliability was calculated to investigate the consistency of the coding tree’s application.

**Table 1:** The most commonly used positive and negative terms to describe Visual Clot.

Positive terms		Negative terms	
Terms	Frequency, n	Terms	Frequency, n
Easy or easier	28	Missing	13
Good	16	Quantitative or quantification	11
Fast or faster	13	Information	9
See a problem	12	Values	8
Interpret quickly	11	Time	8
Simple	9	Fibrinogen	6
Understand	9	Numbers	6
Visual	8	Hyperfibrinolysis	6
Interpretation	7	Less	5
Intuitive	7	Confusing	4
At a glance	7	Flashing	4
Overview	7	Simplified	3

#### Part II: Survey

The literature states that quantitative data can help generalize and confirm specific observations found in qualitative research [29-32]. For the subsequent survey, we defined 5 statements based on the previously identified themes. The same group of interviewed anesthesiologists was asked to rate them on 5-point Likert scales in a questionnaire created using Google Forms (Alphabet Inc). Participants were informed that the survey takes only a few minutes to complete, participation is voluntary, and no compensation is offered. The translated announcement of the survey invitation is displayed in Multimedia Appendix 3. The data collection was finished 3 weeks after the questionnaire was sent.

### Statistical Analysis

The interview data analysis and figures were made using Microsoft Word and Excel (Microsoft Corp). We present the number of statements and their percentage distribution in the identified themes.

To define the interrater reliability of the coding template, we calculated Fleiss’ Kappa using R (version 4.0.5; R Foundation for Statistical Computing). We calculated every statement's median and IQR for the survey analysis. We used the Wilcoxon signed rank test to determine the difference between the median and neutral answers. Statistical significance was indicated as *P*<.05.

## Results

### Study and Participant Characteristics

Detailed information on the study and participants is provided in Table 2. Residents and nurses were the dominant participants in the interviews. The most experienced participant had 33 years of experience in anesthesia. The least experienced had less than 1 year. Residents and nurses also dominated the survey.

**Table 2:** Study and participant characteristics.

Characteristics			Values
**Study characteristics**			
	Total number of interviewed participants, n		77
	Total number of participants completed the survey, n		23
**Participant characteristics**			
	**Interview participants**		
		Staff physicians, n (%)	8 (10.4)
		Residents, n (%)	35 (45.5)
		Anesthesiology nurses, n (%)	34 (44.2)
		Anesthesia experience in years, median (IQR)	8 (3-10)
		Number of ROTEM^a^ interpretations per year, median (IQR)	26 (5-41)
	**Survey participants**		
		Staff physicians, n (%)	7 (30.4)
		Residents, n (%)	8 (34.8)
		Anesthesiology nurses, n (%)	8 (34.8)

^a^ROTEM: rotational thromboelastometry.

### Part I: Qualitative Analysis of Interview Answers

#### Word Count Analysis

The most frequently used words and word combinations used to describe the advantages of Visual Clot were: easier or easy (26/77, 33.8%), interpret or interpretation (23.4%, 18/77 participants), quick or quickly (19.5%, 15/77 participants), visual, visualize, visualized, or visualization (19.5%, 15/77 participants), good (16.9%, 13/77 participants), faster or fast (15.6%, 12/77 participants). In the group of statements describing the limitations of Visual Clot, the words and word groups most frequently used were: ROTEM (23.4%, 18/77 participants), missing (information or values or numbers; 16.9%, 13/77 participants), quantitative or quantification (16.9%, 13/77 participants). Table 1 visually represents the most commonly used words in positive and negative perceptions.

#### Coding Tree

Figure 2 shows the generated coding tree, including 2 main domains and 9 themes. The interrater reliability of the tree raters was 0.856 (95% CI 0.831-0.880), indicating almost perfect agreement [33].

#### Statements Describing Visual Clot

Table 3 demonstrates examples of statements assigned to particular subtopics with participant counts and percentages.

A total of 4 comments were defined as positive but not assigned to any themes. There was 1 such statement in the negative group. A total of 19 comments were not assigned to any theme and were described as noncodable.

**Table 3:** Statements examples assigned to particular domain and subtopics with participant count and percentages.

Major domain and subtopics			Examples
**Positive statements describing Visual Clot (179/319, 56.1%)**			
	Positive design features (63/295, 21.4%)	“Very simplified” (Participant 13). “Visual presentation” (Participant 21). “Tells figuratively what to do” (Participant 26).	
	Positive usability features (30/295, 10.2%)	“It can be perfectly integrated in the clinic” (Participant 24). “A good tool to get an overview” (Participant 32). “You can see at-a-glance what is missing” (Participant 70).	
	Intuitive and easy to learn (61/295, 20.7%)	“Directly applicable and does not require long training” (Participant 5). “Very intuitive” (Participant 75). “Easy to understand” (Participant 76).	
	Time saving (21/295, 7.1%)	“2-3 seconds to a quick overview” (Participant 22). “Immediate detection of the problem” (Participant 5). “Quick to interpret” (Participant 27).	
**Negative statements describing Visual Clot (113/319, 35.4%)**			
	Design problems (23/295, 7.8%)	“Confusing, blinking” (Participant 57). “Quality of hyperfibrinolysis difficult to demonstrate” (Participant 22). “Not everything you see is relevant” (Participant 24).	
	Usability problems (12/295, 4.1%)	“Quantity of change is not visible” (Participant 2). “You have to know the pictures first” (Participant 30). “No prioritization possible” (Participant 76).	
	Incompleteness: a lack of a quantitative component (62/295, 21.0%)	“Also values that are in normal range—is it close to the limit or not?” (Participant 54). “No graduation ‘all or nothing’” (Participant 60). “Exact quantification not possible” (Participant 4).	
	Need for training (15/295, 5.1%)	“Not yet established” (Participant 29). “Needs to get used to it” (Participant 45). “Needs habituation (not used by default yet)” (Participant 50).	
**Combination: Visual Clot and ROTEM^a^ (8/295, 2.7%)**			
			“Would be nice to have it together with ROTEM” (Participant 50). “Ideal solution if could be combined with ROTEM” (Participant 49). “Combination of both necessary” (Participant 74).

^a^ROTEM: rotational thromboelastometry.

#### Positive Statements Describing Visual Clot

##### Positive Design Features

Most comments were made on this topic, emphasizing that a “pictorially summarized” (participant 2) and “visually appealing” (participant 3) data presentation allows one to see “the relevant ROTEM information at-a-glance” (participant 9). Such a design supports health care professionals in making clinical decisions. It is essential in emergencies because the actual coagulation status is immediately visible (participant 14) and it is instantly apparent which hemostasis components are missing (participant 12).

##### Positive Usability Features

Visual Clot is “a good tool for broad application,” stated participant 72. It enables “pre-interpretation of the complex information” (participant 3) and focuses “on the essential” (participant 65). The benefits of the Visual Clot in urgent situations were also highlighted: the technology is “very good for emergencies,” stated participant 13.

##### Intuitive and Easy to Learn

As in the previous study [24], the Visual Clot was also described here as intuitive and easy to learn. “Very intuitive, short time needed to understand it,”—pointed out participant 6. It was underlined that visualizations provided by the Visual Clot are “quickly recognizable even by untrained persons or with little knowledge of coagulation” (participant 13).

##### Time Saving

The Visual Clot provides an “overview at-a-glance,” as participant 76 said. “I immediately saw what was missing,” stated participant 16. These features lead to quicker diagnosis—“focus is faster on the problem”—as participant 46 said, and thus to faster initiation of treatment.

#### Negative Statements Describing Visual Clot

##### Design Problems

Several ideas that could potentially enhance Visual Clot's design were identified. Participant 47 pointed out that the presentation of platelets and fibrinogen are similar, and thus it is difficult to distinguish. Participant 48 also agreed: “I did not notice that platelets were missing because it was white and dashed like fibrinogen.” Some participants found that the Visual Clot is too dynamic—too much movement on the screen, which can lead to distraction and make the interpretation of the results difficult “Even if coagulation status is fine, everything is moving, and you can poorly differentiate what is missing” (participant 53), “moves too much, even if everything is fine—distraction” (participant 55).

##### Usability Problems

Visual Clot is “confusing at the beginning”—stated participant 31 and added that it is “difficult to use without routine.” Visual Clot provides “too much information at once,”—participant 53 pointed out.

##### Incompleteness: Lack of a Quantitative Component

The central Visual Clot aspect criticized was the technology's incompleteness in terms of lacking a quantitative component. Several participants stated that the Visual Clot is “not precise” (participant 1), which can be explained in the words of participants 53 and 9, respectively, who said that in the Visual Clot “quantitative is missing” and that one “can get more information with the ROTEM.”

##### Need for Training

The main point identified in the participants’ opinions on this topic was the lack of experience working with this technology and that it is a very new tool not yet established in clinical practice.

##### Combination of Visual Clot and ROTEM

Several participants said they could benefit from combining the Visual Clot and ROTEM when interpreting coagulation assays. “A combination of Visual Clot and ROTEM would be perfect,” pointed out participant 19, while participant 74 said, “a combination of both is necessary.” There was no difference in positive and negative statements based on participants' specialty or level of experience.

### Part II: Analysis of Statements Assessed in the Survey

**Figure 3:**
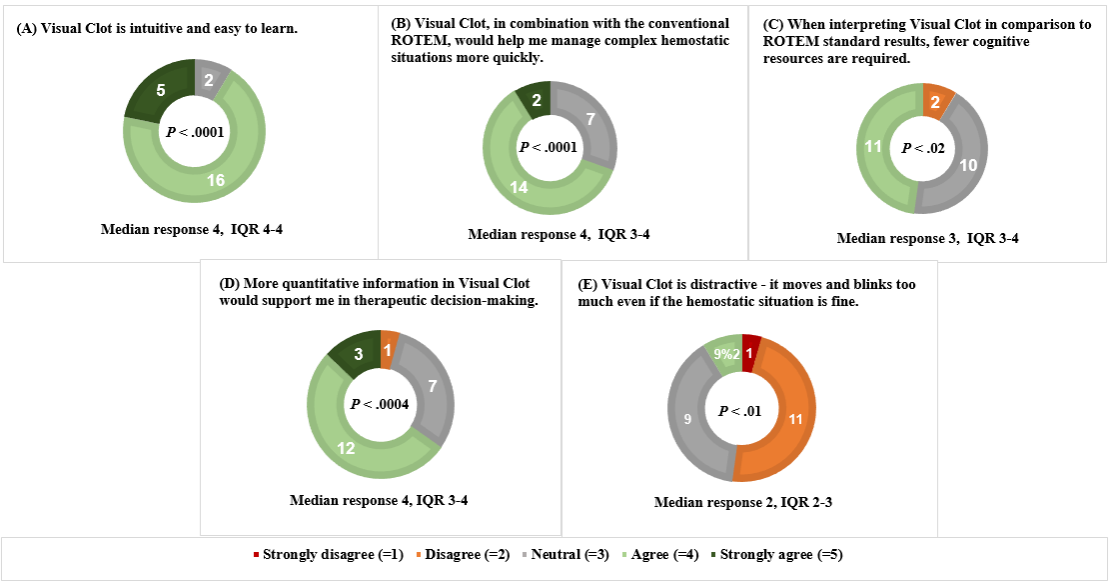
Pie charts presenting survey results with the number of participants who chose a particular category (N=23). ROTEM: rotational thromboelastometry.

Figure 3 shows the detailed evaluation of the statements rated in the survey.

All sample medians differed statistically significantly from neutral (*P*<.05). The number of participants in the quantitative part of the evaluation differs from the qualitative part because not all participants completed the questionnaire. The results are presented as medians and IQR. *P* values are provided to indicate a statistically significant difference between the median of the sample and the neutral value.

## Discussion

### Principal Findings

This mixed qualitative-quantitative study analyzed the perceptions of anesthesiology personnel regarding Visual Clot—a new situation-awareness and user-oriented visualization technology for viscoelastic hemostatic resuscitation—after the high-fidelity simulation study. User perceptions enable us to identify the positive aspects of the technology and reveal the potential for improvement in the future. After computer-based studies, this is the first time that Visual Clot has been evaluated in a high-fidelity simulation study, a validated process for testing a noncertified product in an environment that closely resembles clinical reality [34,35].

The principal findings demonstrate that the design features of Visual Clot have received the most positive comments. As in the previous computer-based Visual Clot study [24], the participants of this high-fidelity simulation study emphasized that the way this technology is designed provides a good overview of the clotting situation and is an additional help in the decision-making process during acute bleeding situations. Further, Visual Clot was described as intuitive and easy to learn. Participants repeatedly mentioned that the results of Visual Clot are quickly recognizable and understandable even by inexperienced clinicians. The main criticism concerned the lack of quantitative information.

Previous Visual Clot studies [22-24,36,37] underline the benefits of additional visualization technology, simplifying standard ROTEM data interpretation [20]. Anesthesia providers using Visual Clot in a high-fidelity simulation study were more likely to correctly administer targeted coagulation therapy and to give the first targeted coagulation product faster. In addition, participants demonstrated greater decision-making confidence with Visual Clot [23]. Moreover, the correctness of the clinical decisions was independent of previous rotational thromboelastometry knowledge and experience.

The superior participants’ performance when working with Visual Clot may be explained by its design supporting the strengths of human sensory perception. The Visual Clot was developed to assist care providers in managing highly complex coagulation situations, presenting the data in an awareness-oriented interface design. The main aim of this design is to convey the information as quickly as possible and with the lowest cognitive effort [25].

Principles of situation awareness-oriented and user-centered design enables effective data management and a comprehensive understanding of what is happening and thus help to stay situationally aware. This concept is essential in many domains, including medicine, where managing complex and dynamic situations is fundamental [38,39].

Its definition breaks down into three separate phases (1) the perception of environmental elements in the current situation within a volume of time and space, (2) understanding their meaning, and (3) their projection in the near future. Based on this, the Visual Clot data are visually represented, preprocessed, and simplified. The results of coagulation parameters are divided into 3 categories: too low, normal, or too high. Such information presentation increases diagnostic confidence, but numeric indicators are needed for precise data analysis and targeted treatment initiation. As previously indicated, the lack of quantitative information is reflected in user responses. It also explains the participants’ considerations that combining Visual Clot and ROTEM would be helpful in clinical decision-making.

Some other technologies based on situation awareness and user-centered design principles include Philips Visual Patient Avatar (Philips) [40], AlertWatch (AlertWatch Inc), Dynamic Lung Panel and PulmoSight (Hamilton Medical AG), HemoSight and Physiology Screen (Mindray Medical International Limited), and Alarm Status Visualizer (Masimo Corp) [41,42].

This study showed user perceptions regarding the new situation awareness-based, user-oriented technology for thromboelastometry data presentation—Visual Clot. It makes us aware of the user’s needs and could help us simplify information processing and decision-making in the future. An integral facet of advancing the technological framework informed by the results of this study lies in the prospect of merging quantitative data into the Visual Clot platform and presenting this merged information in a consolidated interface. This concerted integration promises to align both quantitative and qualitative data to provide a more complete and accurate representation of prevailing conditions. This integration can be achieved in a variety of ways, including the direct overlay of numerical values onto the Visual Clot visualization, or the parallel juxtaposition of a complementary graphical representation alongside the numerical data set.

### Strengths and Limitations

This study has several strengths and limitations. The interview part of the study has the typical limitations of qualitative research. The findings of qualitative analysis cannot be extrapolated to larger populations with the same certainty as quantitative results because the findings are on the subjective basis and not tested for statistical significance [43]. However, the quantitative survey helped to provide greater insight into the importance of the main themes identified. Moreover, the interviewed participants were selected according to their availability in the clinical praxis and not randomly.

Furthermore, the number of participants in the survey was lower than in the interviews because not all participants in the simulation study completed the survey. Finally, it is a single-center study performed in a university hospital with high care standards in Europe. User perceptions may vary across diverse clinical settings in different parts of the world.

### Conclusions

After previous studies investigating user perceptions of Visual Clot in computer-based simulation studies, this is the first study to analyze the user perceptions of Visual Clot in a high-fidelity simulation—the intermediate step between computer-based simulation studies in a laboratory and real-life use. In this study, Visual Clot appeared to be a well-accepted additional tool supporting health care professionals working with ROTEM. Based on participants’ perceptions, user-centered and situation awareness-oriented design, as shown in Visual Clot, can simplify the presentation of complex information and thus make critical decision-making quicker and more efficient. The benefits of this technology have been particularly highlighted in emergencies and even for care providers with little experience in coagulation management. Participants described Visual Clot as intuitive and easy to learn. The lack of a quantitative component has been identified as a significant limitation. These findings highlight the advantages of Visual Clot and its potential for improvement may help further develop this and other situation awareness-based technologies.

## Appendix

Multimedia Appendix 1 Educational Visual Clot video.

Multimedia Appendix 2 Complete translated field notes of participant interviews.

Multimedia Appendix 3 Translated announcement of the survey invitation.

## Abbreviations

NASA: National Aeronautics and Space Administration
ROTEM: rotational thromboelastometry

## Data Availability

The data sets used and analyzed during this study are available from the corresponding author on reasonable request.
