# Supplementary material for: User Perceptions of Visual Clot in a High-Fidelity Simulation Study: Mixed Qualitative-Quantitative Study
Source: JMIR Hum Factors. 2024 Jan 11;11:e47991. doi: 10.2196/47991 (PMC10811569; doi:10.2196/47991)
Supplement: Multimedia Appendix 2 [file humanfactors_v11i1e47991_app2.pdf]

Participant 1

POSITIVE: What did you LIKE about the VC?

[If woman understood it - well.]

NEGATIVE: What did you *DISLIKE* about the VC?

[Needed a moment more to understand it.] [No values (not precise).]

Participant 2

POSITIVE: What did you LIKE about the VC?

[Self-explanatory, you just have to look at it and you can use it.Even if you have never seen it before.] [Anyone can use it.] [Pictorially summarized ROTEM information.] [Still need to decide how much to give.] [Find it pretty cool.] {Simple straightforward information.] [It is a bit like using the BIS as an additive aid to the EEG curve.]

NEGATIVE: What did you *DISLIKE* about the VC?

[Would need ROTEM at the same time, possibly before ROTEM to see exactly how much you need.] [Quantity of change not visible.]

Participant 3

POSITIVE: What did you LIKE about the VC?

[Pre-interpretation of the complex information.] [Dynamic] [simple] [visually appealing.]

NEGATIVE: What did you *DISLIKE* about the VC?

[Boundary decisions possibly pre-decided.] [ROTEM would offer more scope for interpretation.]

Participant 4

POSITIVE: What did you <u>LIKE</u> about the VC?

[Much faster.] [Search what is missing.]

NEGATIVE: What did you *DISLIKE* about the VC?

[Exact quantification not possible.] [Could lead to over/under dosing.]

Participant 5

POSITIVE: What did you LIKE about the VC?

[Immediate detection of the problem.] [Directly applicable and does not require long training.] [No good education needed to interpret VC correctly.] [Would be good for everyday life especially in stressful situations where the problem must be recognized quickly.]

NEGATIVE: What did you *DISLIKE* about the VC?

[No quantitative data.] [Could possibly be programmed in addition (e.g. for fibrinogen).]

Participant 6

POSITIVE: What did you LIKE about the VC?

[Very intuitive, short time needed to understand it.] [Even e.g. sub-assistants and student nurses can understand it an interpret it in a hectic situation and pass it on correctly.]

NEGATIVE: What did you *DISLIKE* about the VC?

[Qualitative] [not so accurate] [for rough 10 minutes great, then missing qualitative values.]

Participant 7

POSITIVE: What did you <u>LIKE</u> about the VC?

[Easy to interpret for everyone.] [For untrained better to use and interpret.]

NEGATIVE: What did you *DISLIKE* about the VC?

[You cannot see the individual values, which could be too simplified for more experienced ROTEM users.]

Participant 8

POSITIVE: What did you <u>LIKE</u> about the VC?

[Easy to interpret for everyone.] [For untrained better to use and interpret.]

NEGATIVE: What did you *DISLIKE* about the VC?

[You cannot see the individual values] [which is simplified for more experienced ROTEM users.]

Participant 9

POSITIVE: What did you LIKE about the VC?

[Great that at a single look the relevant «ROTEM signs» are displayed.] [The interpretation was much easier than with the conventional ROTEM.] [I did not have the feeling to miss something.]

NEGATIVE: What did you *DISLIKE* about the VC?

[There is a loss of information with the VC from time to time.] [If I have time, I can get more information with the ROTEM.]

Participant 10

POSITIVE: What did you LIKE about the VC?

[For the shock room, if it is acute, the ROTEM for long operations more suitable due to more differentiated data.]

NEGATIVE: What did you *DISLIKE* about the VC?

Nothing

Participant 11

POSITIVE: What did you LIKE about the VC?

[Interesting] [easier therapy decisions, what needs to be replaced, what does not.] [For people who don’t deal with it much.]

NEGATIVE: What did you *DISLIKE* about the VC?

[Nothing.]

Participant 12

POSITIVE: What did you LIKE about the VC?

[It goes to the eye what is missing] [you see immediately what is missing.] [Requires less time to install] [easier handling.]

NEGATIVE: What did you *DISLIKE* about the VC?

[Numbers missing how much to give.] [How does it continue in the progress, you look then no more on it.]

Participant 13

POSITIVE: What did you LIKE about the VC?

[Very good for emergency situations.] [Very simplified.] [One does not need to know much about coagulation] [quickly recognized even by untrained persons or with little knowledge of coagulation.]

NEGATIVE: What did you *DISLIKE* about the VC?

[Does not give much] [simplified.]

Participant 14

POSITIVE: What did you <u>LIKE</u> about the VC?

[Also very good] [you can see directly the situation.]

NEGATIVE: What did you *DISLIKE* about the VC?

[It is not known enough.]

Participant 15

POSITIVE: What did you LIKE about the VC?

[Easy to interpret.]

NEGATIVE: What did you *DISLIKE* about the VC?

[Information gets lost] [less precise.]

Participant 16

POSITIVE: What did you <u>LIKE</u> about the VC?

[Much easier.] [I immediately saw what was missing.] [For me as a new person in the business it is faster to understand.]

NEGATIVE: What did you *DISLIKE* about the VC?

[Nothing.] [But is it then more inaccurate possibly?]

Participant 17

POSITIVE: What did you LIKE about the VC?

[Good design.] [Very easy to understand] [easier than ROTEM especially for beginners.]

NEGATIVE: What did you *DISLIKE* about the VC?

[I think it depicts a coagulation disorder in less detail.]

Participant 18

POSITIVE: What did you LIKE about the VC?

[Unfortunately, I have not been able to make a simulation with the VC.] [What is missing is recognized directly.] [The interpretation is learned quickly and easily.]

NEGATIVE: What did you *DISLIKE* about the VC?

[No quantitative information (no exact information on the quantity that should be substituted).]

Participant 19

POSITIVE: What did you LIKE about the VC?

[Funny, especially the hyperfibrinolysis. The rest I have blurred in memory.]

NEGATIVE: What did you *DISLIKE* about the VC?

[I had too short time to deal with the VC to see the missing products.] [A combination of VC and ROTEM would be perfect.]

Participant 20

POSITIVE: What did you LIKE about the VC?

[Much more intuitive] [you can see what is going on at first sight][Even if you are not trained.]

NEGATIVE: What did you *DISLIKE* about the VC?

[No timeline] [no scale to estimate.]

Participant 21

POSITIVE: What did you LIKE about the VC?

[Easy to understand.] [Visual presentation.]

NEGATIVE: What did you *DISLIKE* about the VC?

[No timeline] [invisible if completed or still running.] [Information-processing from raw data to image not clearly visible (data is lost).] [Questioning because of measures possibly not equally good because (too) simple (e.g. Periplex in CHD patient has to be carefully weighed up).]

Participant 22

POSITIVE: What did you LIKE about the VC?

[Everything at a glance with five icons.] [2-3 seconds to a quick overview.]

NEGATIVE: What did you *DISLIKE* about the VC?

[Quality of hyperfibrinolysis difficult to demonstrate.]

Participant 23

POSITIVE: What did you LIKE about the VC?

[Easier to interpret when having less experience with ROTEM.] [In the last scenario, I only had Visual Clot. It was quickly clear what was missing]

NEGATIVE: What did you *DISLIKE* about the VC?

[Very new.] [It is hard to judge in one day.] [I could have done it with the conventional ROTEM as well.]

Participant 24

POSITIVE: What did you LIKE about the VC?

[Very intuitive, self-explanatory.] [Can be perfectly integrated in to the clinic.]

NEGATIVE: What did you *DISLIKE* about the VC?

[Not everything you see is relevant (the 3 big EC in the background are disturbing my perception, distracting me).]

Participant 25

POSITIVE: What did you LIKE about the VC?

[Very intuitive] [well understandable] [needs not much training.]

NEGATIVE: What did you *DISLIKE* about the VC?

[Less distinguished (only yes/no).]

Participant 26

POSITIVE: What did you LIKE about the VC?

[Yes or no principle.] [Tells figuratively what to do.]

NEGATIVE: What did you *DISLIKE* about the VC?

[See above (yes/no principle), figuratively indicating what to do without knowing the tendency).]

Participant 27

POSITIVE: What did you LIKE about the VC?

[Important results.] [First overview faster] [simple] [quick to interpret.]

NEGATIVE: What did you *DISLIKE* about the VC?

[Quantitative assessment is missing.]

Participant 28

POSITIVE: What did you <u>LIKE</u> about the VC?

[Easier to read.] [Graphical representation.] [When you grasp it, you can distinguish it quickly.]

NEGATIVE: What did you *DISLIKE* about the VC?

[Progress not visible (e.g. fibrinolysis from when) - no trend visible.] [Ideal: would suggest therapy (2g fibrinogen or 4g e.g.).]

Participant 29

POSITIVE: What did you LIKE about the VC?

[Very appealing for beginners] [quick to learn.]

NEGATIVE: What did you *DISLIKE* about the VC?

[Not yet established.] [Non-quantitative.]

Participant 30

POSITIVE: What did you LIKE about the VC?

[Visual.] [Fast/simple diagnosis with little previous knowledge.]

NEGATIVE: What did you *DISLIKE* about the VC?

[You have to know the pictures first.] [Quantitative aspects are missing.]

Participant 31

POSITIVE: What did you LIKE about the VC?

[Flashing pathological «values» helpful (if you know them).]

NEGATIVE: What did you *DISLIKE* about the VC?

[Difficult to use without routine (a bit confusing at the beginning).] [Not yet established.]

Participant 32

POSITIVE: What did you LIKE about the VC?

[Overall a good thing to get an overview] [good approach and overview with one picture (and not 4).]

NEGATIVE: What did you *DISLIKE* about the VC?

[You are used to ROTEM, you do not have an assessment yet, you have to look at the legend] [not as concise as ROTEM.]

Participant 33

POSITIVE: What did you LIKE about the VC?

[Fibrin visualized well] [platelets also visualized well], [bleeding also logical.]

NEGATIVE: What did you *DISLIKE* about the VC?

[VC reinterpreted into ROTEM.] [Hyperfibrinolysis not intuitive.]

Participant 34

POSITIVE: What did you LIKE about the VC?

[Very clear what is missing and what is there] [better for understanding if you get it explained.] [Helpful if you like to learn with pictures] [especially the moving pictures are helpful and the legend.]

NEGATIVE: What did you *DISLIKE* about the VC?

[Nothing.]

Participant 35

POSITIVE: What did you LIKE about the VC?

[Much more intuitive than ROTEM] [free of numbers, because first question is: «Should we give something? And not how much.] [Dynamic visualization (blinking, dripping, milling).] [Has a legend.]

NEGATIVE: What did you *DISLIKE* about the VC?

[Not yet so much concerned with it, needs it in everyday life.]

Participant 36

POSITIVE: What did you <u>LIKE</u> about the VC?

[Very cool, works even faster.] [Easier to master without experience.] [Simple.] [In emergency situations, it cannot be too simple.]

NEGATIVE: What did you *DISLIKE* about the VC?

[Nothing.]

Participant 37

POSITIVE: What did you <u>LIKE</u> about the VC?

[It is moving (makes sense).]

NEGATIVE: What did you *DISLIKE* about the VC?

[One does not know yet/not much experience with it.]

Participant 38

POSITIVE: What did you <u>LIKE</u> about the VC?

[Easier than ROTEM.] [At the very beginning of the education it would have been easier with VC.]

NEGATIVE: What did you *DISLIKE* about the VC?

[First experience today with VC.]

Participant 39

POSITIVE: What did you <u>LIKE</u> about the VC?

[Visual.] [Easier interpretable.]

NEGATIVE: What did you *DISLIKE* about the VC?

[Nothing.]

Participant 40

POSITIVE: What did you LIKE about the VC?

[Much easier to see at a glance what the problem is.][You do not have to look at questions and evaluate them.] [More reliable in diagnosis.]

NEGATIVE: What did you *DISLIKE* about the VC?

[Nothing.]

Participant 41

POSITIVE: What did you LIKE about the VC?

[Simpler than the conventional traces.][Would be easier to understand and react to immediately than with what we have now.]

NEGATIVE: What did you *DISLIKE* about the VC?

[Nothing.]

Participant 42

POSITIVE: What did you LIKE about the VC?

[Fibrinogen can be seen quickly if it is missing] [also platelets.]

NEGATIVE: What did you *DISLIKE* about the VC?

[Whole or no principle, no tendency (e.g. if fibrinogen is scarce, but it is still displayed normal).] [Also needs time to think and gain experience.] [That with heparin the symbol is added, one must rethink, uncertain when hyperfibrinolysis is added (must one look at it again?) still unclear).] [How much VC has already run, i.e. at which point in time you are (at the ROTEM it is displayed).]

Participant 43

POSITIVE: What did you LIKE about the VC?

[Clearly structured] [you can see quickly what is missing from the six things.] [Qualitatively good.]

NEGATIVE: What did you *DISLIKE* about the VC?

[Quantitatively poorly evaluable.]

Participant 44

POSITIVE: What did you <u>LIKE</u> about the VC?

[Well-structured.] [You can imagine how it looks inside the body and it is easier to make decisions.]

NEGATIVE: What did you *DISLIKE* about the VC?

[You do not know how much to substitute of what.] [Time course not visible with VC.]

Participant 45

POSITIVE: What did you <u>LIKE</u> about the VC?

[Easy to see.]

NEGATIVE: What did you *DISLIKE* about the VC?

[Needs to get used to it.]

Participant 46

POSITIVE: What did you LIKE about the VC?

[Easier interpretation for beginners.] [Well-structured.] [Focus is faster on the problem.]

NEGATIVE: What did you *DISLIKE* about the VC?

[Interpretation would be easier with numbers (for the more advanced).]

Participant 47

POSITIVE: What did you LIKE about the VC?

[Easier interpretation (if you know it).] [Faster to learn.]

NEGATIVE: What did you *DISLIKE* about the VC?

[Color of platelets and hyperfibrinolysis are similar (more difficult to distinguish).] [Needs as much manpower as ROTEM.]

Participant 48

POSITIVE: What did you LIKE about the VC?

[You can look at it and know where the problem is.]

NEGATIVE: What did you *DISLIKE* about the VC?

[Did not notice that platelets were missing because it was white and dashed like fibrinogen.]

Participant 49

POSITIVE: What did you LIKE about the VC?

[Good for visual learning type because it summarizes a part of the clotting system compactly and presents it better with an animation.] [Could interpret scenarios better with it.] [Ideal solution if can be combined with ROTEM.] [Presentation (aesthetic).]

NEGATIVE: What did you *DISLIKE* about the VC?

[Too simple to determine everything that ROTEM would show.]

Participant 50

POSITIVE: What did you LIKE about the VC?

[Visualization more simple.]

NEGATIVE: What did you *DISLIKE* about the VC?

[Needs habituation (not used by default yet)] [would be nice to have it together with ROTEM.]

Participant 51

POSITIVE: What did you LIKE about the VC?

[Legend is helping to identify the problem faster.]

NEGATIVE: What did you *DISLIKE* about the VC?

[Like a game.]

Participant 52

POSITIVE: What did you LIKE about the VC?

[Visual it is recognized faster, one notices faster what the problem is.]

NEGATIVE: What did you *DISLIKE* about the VC?

[No numbers.]

Participant 53

POSITIVE: What did you LIKE about the VC?

[Beautifully colored] [you can see everything at a glance.]

NEGATIVE: What did you *DISLIKE* about the VC?

[Quantitative is missing.] [Even if it is fine everything is moving and then you can poorly differentiate what is missing or not.] [In case of hyperfibrinolysis and fibrin is missing. What is the cause? (is fibrinogen missing or is it hyperfibrinolysis? Because fibrin was dashed – not clearly visible).] [Too much information at once][not necessarily faster than with ROTEM.] [Do you have forgotten something when everything is flashing, a bit confusing.]

Participant 54

POSITIVE: What did you <u>LIKE</u> about the VC?

[If you get used to it, it is quite easy to interpret, especially with legend] [descriptive tool] [easy to learn, you do not have to learn it, but get used to it.] [Like a flow chart.]

NEGATIVE: What did you *DISLIKE* about the VC?

[No absolute values] [also values that are in normal range - is it close to the limit or not?]

Participant 55

POSITIVE: What did you <u>LIKE</u> about the VC?

[Better structured than ROTEM.]

NEGATIVE: What did you *DISLIKE* about the VC?

[Less accurate than ROTEM.] [You do not see any progression.] [Moves too much (even if everything is fine, distraction).]

Participant 56

POSITIVE: What did you LIKE about the VC?

[All information displayed in a summarized form (instead of 4 different graphics).]

NEGATIVE: What did you *DISLIKE* about the VC?

[A bit confusing at first sight (because everything together).] [No course.]

Participant 57

POSITIVE: What did you LIKE about the VC?

[Nothing.]

NEGATIVE: What did you *DISLIKE* about the VC?

[Confusing, blinking.] [No numbers.] [Unfamiliar.] [Would be easier/better if there was already the "solution"/therapy/suggestion given (e.g. …give/consider) (as an alternative to the VC)]

Participant 58

POSITIVE: What did you <u>LIKE</u> about the VC?

[It is good to try to visualize it.] [More attractive at first sight.]

NEGATIVE: What did you *DISLIKE* about the VC?

[Still unknown.]

Participant 59

POSITIVE: What did you LIKE about the VC?

[Droplets symbolize that something is not good (gaze diagnosis).]

NEGATIVE: What did you *DISLIKE* about the VC?

[Missed introduction.] [Not used to working with it, ROTEM preferred/used to it.] [Needs a lot effort to get it into the brain.] [Only VC would not be good/sufficient and parallel with ROTEM one would look only at the ROTEM.]

Participant 60

POSITIVE: What did you LIKE about the VC?

[Very intuitive.] [Needs little practice and prior knowledge] [“realistic” visualization (Clot in EM)]

NEGATIVE: What did you *DISLIKE* about the VC?

[No graduation («all or nothing»).]

Participant 61

POSITIVE: What did you LIKE about the VC?

[Well structured (at a glance).] [Needs less to understand.]

NEGATIVE: What did you *DISLIKE* about the VC?

[Hectic (by flashing).]

Participant 62

POSITIVE: What did you LIKE about the VC?

[Very self-explanatory] [has a legend.] [Sees with a second what is missing.]

NEGATIVE: What did you *DISLIKE* about the VC?

[There is no quantitative statement, just if something is missing or not.] [That you do not have it in clinical practice yet.]

Participant 63

POSITIVE: What did you LIKE about the VC?

[Nice visual aspect] [Harvesters are good/problem is quickly detected] [drops show bleeding.] [It is easier to understand]

NEGATIVE: What did you *DISLIKE* about the VC?

[No numbers] [Too much information on a small screen.] [but as a tool itself not enough – in combination with ROTEM not bad.]

Participant 64

POSITIVE: What did you LIKE about the VC?

[Good initial overview.] [You quickly see what the problem is. Harvester/fibrin flashing/…]

NEGATIVE: What did you *DISLIKE* about the VC?

[For more precise things a bit scarce, not displayable, strict cut-off value, no grey area.][With even more screens, would blink even more (sensory overload).]

Participant 65

POSITIVE: What did you LIKE about the VC?

[Focused on the essential.]

NEGATIVE: What did you *DISLIKE* about the VC?

[No values displayed.]

Participant 66

POSITIVE: What did you LIKE about the VC?

[Easier to recognize.]

NEGATIVE: What did you *DISLIKE* about the VC?

[Nothing.]

Participant 67

POSITIVE: What did you <u>LIKE</u> about the VC?

[Easy interpretation even for beginners (roadworthy).]

NEGATIVE: What did you *DISLIKE* about the VC?

[Heparin could also be flashing (otherwise you do not notice that it is there).]

Participant 68

POSITIVE: What did you <u>LIKE</u> about the VC?

[Good overview of coagulation disorders.] [Rather fast.] [Interpretable for everyone.]

NEGATIVE: What did you *DISLIKE* about the VC?

[No absolute values.] [Relative small.]

Participant 69

POSITIVE: What did you <u>LIKE</u> about the VC?

[Quickly a visual diagnosis, in one glance everything in front of you.] [You see it directly.]
[Suitable for all professions, you do not have to «learn» it.]

NEGATIVE: What did you *DISLIKE* about the VC?

[Limited because you can not see any numbers] you can not even see how narrow it is.]
[Heparin is not flashing.]

Participant 70

POSITIVE: What did you <u>LIKE</u> about the VC?

[You can see immediately at a glance what is missing.]

NEGATIVE: What did you *DISLIKE* about the VC?

[Heparin does not flash and is therefore less noticeable (because it is additional).]

Participant 71

POSITIVE: What did you LIKE about the VC?

[You can see pretty fast the main problem.] [Needs less training (than ROTEM).]

NEGATIVE: What did you *DISLIKE* about the VC?

[Lack of quantification (MCF).]

Participant 72

POSITIVE: What did you <u>LIKE</u> about the VC?

[Needs no practice, no interpretation.] [Good tool for wide application.]

NEGATIVE: What did you *DISLIKE* about the VC?

[If you are used to ROTEM, VC seems almost too simple, can not read out so much.] [Provides an interpretation (e.g. platelets missing, from ROTEM only to derive, but not directly).]

Participant 73

POSITIVE: What did you LIKE about the VC?

[Visualization simple] [well mapped] [color coded] [quick to identify.] [Requires less resources/training.]

NEGATIVE: What did you *DISLIKE* about the VC?

[Background dark (attention not «aroused»).]

Participant 74

POSITIVE: What did you LIKE about the VC?

[You could immediately see what is primarily the problem.]

NEGATIVE: What did you *DISLIKE* about the VC?

[No quantification (how much).] [Combination of both necessary.]

Participant 75

POSITIVE: What did you LIKE about the VC?

[Very intuitive.]

NEGATIVE: What did you *DISLIKE* about the VC?

[No quantitative information.]

Participant 76

POSITIVE: What did you LIKE about the VC?

[Overview at a glance] [easy to understand.] [Quickly learnable.]

NEGATIVE: What did you *DISLIKE* about the VC?

[Only black and white (either it is missing or not)] [no prioritization possible][Difference whether fibrin is at 7 or 6 or 2 – and how fast and how much fibrinogen should be given.]

Participant 77

POSITIVE: What did you LIKE about the VC?

[I like the picture a lot, clear and understandable.] [You can find mistakes/what is the problem faster.] [It is simple, has a structure.] [Better for emergencies, can focus better (are strokes yellow or white/flashing).] [Well recognizable even without glasses.] [Very good also if you can combine it with ROTEM.]

NEGATIVE: What did you *DISLIKE* about the VC?

[Would have to read through it again.]

Translated with www.DeepL.com/Translator (free version)
