# Supplementary material for: User Perceptions of Visual Clot in a High-Fidelity Simulation Study: Mixed Qualitative-Quantitative Study
Source: JMIR Hum Factors. 2024 Jan 11;11:e47991. doi: 10.2196/47991 (PMC10811569; doi:10.2196/47991)
Supplement: Multimedia Appendix 3 [file humanfactors_v11i1e47991_app3.pdf]

Dear colleagues

Although the Patient Blood Management (ROTEM and Visual Clot) simulation study was a while ago, we hope you remember the user-centered technology - Visual Clot.

Our research team is engaged in developing a user-centered and situational working environment - your opinion and needs are the basis for this.
Therefore, we have created a short survey on Visual Clot that reflects your views on different aspects of using this technology.

Link to the survey (duration max. 2 min):
xxx

Best regards and thank you for your contribution to the development of user-centered technologies that support us in life-saving decision-making.

Your research team

Translated with www.DeepL.com/Translator (free version)
